# Pancreatic pseudocyst mimicking a left kidney abscess: a case report

**DOI:** 10.1186/s13256-023-03957-3

**Published:** 2023-05-31

**Authors:** Mohamed Anouar Madani, Yassine Ouannes, Kays Chaker, Mahdi Marrak, Yassine Nouira

**Affiliations:** Department of Urology, LA RABTA Hospital, University of TUNIS EL MANAR, Tunis, Tunisia

**Keywords:** Pancreatic prerenal pseudocyst, Kidney, Abscess, Case report

## Abstract

**Background:**

Pancreatic pseudocyst are fluid filled sacs that develop as a result of dissection of pancreatic enzyme tissue. While most commonly found near the pancreas, they can also rarely occur in other areas such as the perirenal region.

**Case presentation:**

This study reports a new case of an infected perirenal pancreatic pseudocyst mimicking a left kidney abscess in a 46-year-old North African patient with history of recurrent acute pancreatitis, who presented with left lumbar region pain and fever. Computed tomography revealed a left perirenal collection that turned out to be an infected pancreatic pseudocyst, The diagnostic was first suspected based on the medical history of the patient and confirmed by biochemical examination detecting a high level of pancreatic enzymes in the computed tomography-guided percutaneous drainage fluid. The patient evolved well after early resuscitation, rapid and effective antibiotic therapy, and computed tomography-guided percutaneous drainage of renal collection.

**Conclusion:**

Pancreatic pseudocyst is an uncommon disorder, which may present at a complicated stage and that must be considered in patients with a history of pancreatitis.

## Introduction

Pancreatic pseudocysts are fluid-filled sacs that form in the vicinity of the pancreas as result of fascial planes dissection by pancreatic enzymes [1].

Diagnosis is based on computed tomography scans (CT).

While peripancreatic location is the most common, ectopic locations such as liver, spleen, and mediastinum are possible. However, perirenal pancreatic pseudocyst are quite rare.

We report an uncommon case of a pancreatic pseudocyst mimicking a left kidney abscess.

## Case presentation

A 46-year-old North African man with history of five hospitalizations in the space of 3 years for hypertriglyceridemia-induced acute pancreatitis presented to our emergency department with epigastric and left lumbar region pain that had been evolving for 10 days (*T* = 0).

On medical interrogation, the patient did not report other comorbidity nor excessive alcoholic consumption or drug abuse. No surgical history was mentioned. The patient had no significant family history and comes from an economically disadvantaged background. He was not adhering to the recommended regimen and was noncompliant with the prescribed hypertriglyceridemia treatment, was tolerating oral, and refused smoking cessation.

The patient’s physical examination showed that the pain was indistinct in nature and radiated to the left flank and left hypochondrium. The patient was febrile with a normal volume pulse. His hemodynamic state was stable. The abdomen was tender upon deep palpation.

White blood cell count was elevated (14.2 × 103/μL), hemoglobin was within normal levels (13.3 g/dL), and C-reactive protein was found to be elevated (298 mg/L, normal values < 6 mg/L). The other blood chemistry levels, in particular serum lipase and amylase, levels liver function tests, and serum creatinine, were within normal range. Bacteriological examination of urine was negative.

**Fig. 1:**
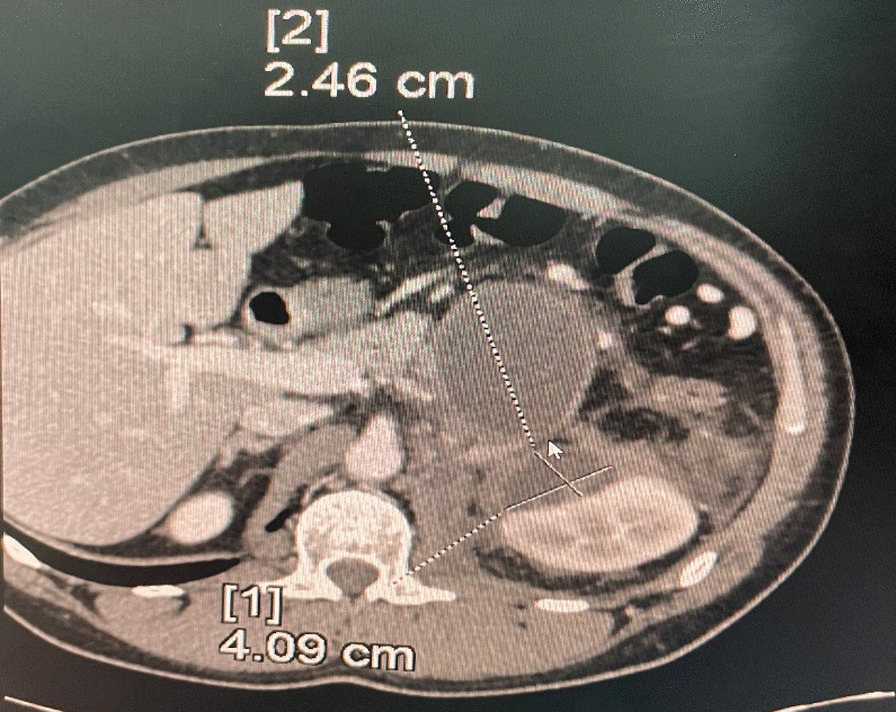
Axial sections of CT scan when the patient first presented to our hospital, showing an important infiltration of the left perirenal and pararenal fat with the presence of a collection of upper pole of the left kidney, which measured 25 × 40 mm and had a liquid density. The collection presented peripheral rim enhancement and the pancreas was within normal size without swelling. The CT also revealed a pancreatic pseudocyst of 68 × 50 mm, which was associated with a discreet dilation of the Wirsung canal

An abdominal computed tomography (*T* = day 1) revealed a 40 × 25 mm left renal abscess and a retroperitoneal 68 × 50 mm pancreatic pseudocyst (Fig. 1).

The patient was admitted to our urology department for the treatment of his renal abscess.

Fluid resuscitation and probabilistic antibiotic treatment with intravenous cefotaxime and gentamicin was administered.

The patient underwent CT-guided percutaneous drainage of renal collection (*T* = day 2).

The fluid was amber colored, and bacteriological and biochemical examination showed high level of amylase (384 UI/L) and turned positive to *Escherichia coli*, which was more in favor of an infected pancreatic pseudocyst.

The patient’s fever subsided under antibiotics, and blood analysis showed a decrease of the white blood cell count and C-reactive protein when the patient completed 15 days of cefotaxime treatment.

**Fig. 2:**
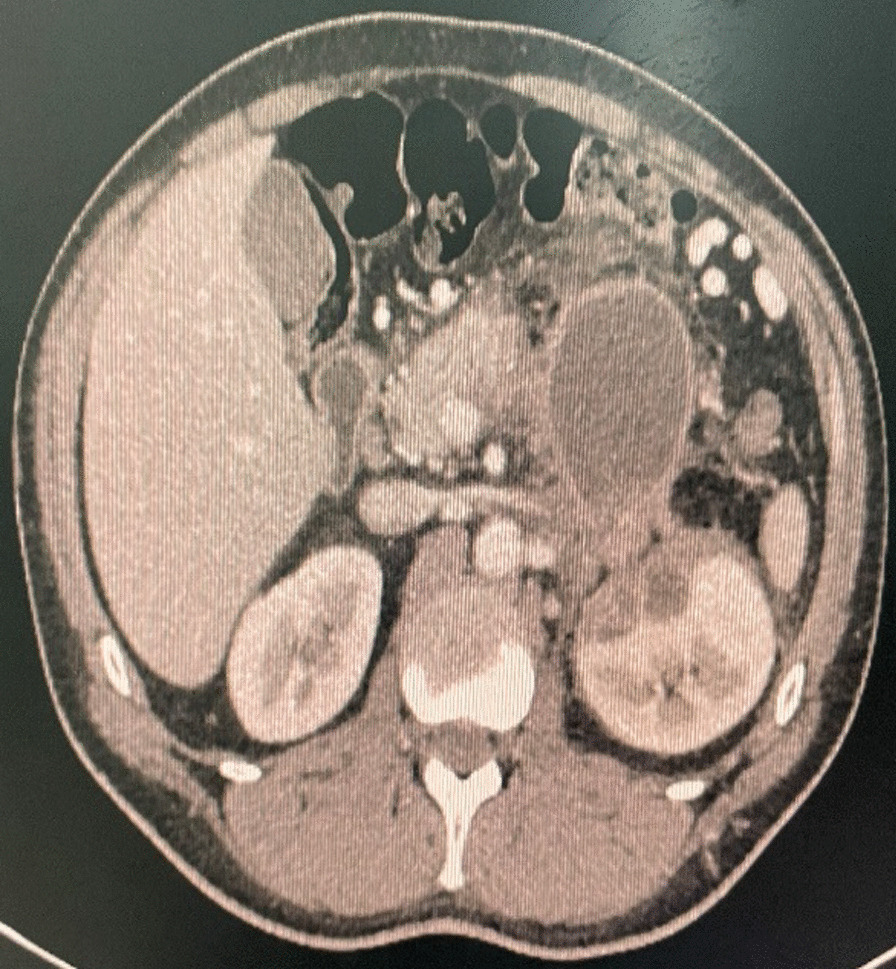
Axial sections of CT scan showing a 20 × 18 mm left renal pseudocyst

A follow-up CT Scan was performed (*T* = Day 10) (Fig. 2), which showed a clear regression of the upper pole renal collection, which measured 20 × 18 mm.

On day 16, he was discharged and transferred to the visceral surgery department to care of the retroperitoneal pseudocyst, with a favorable evolution.

A follow-up ultrasound was performed after 1 month showing total regression of all pseudocysts.

## Discussion

Pancreatic pseudocysts is a rare, benign condition that can complicate up to 10% of cases of acute pancreatitis [2].

Pancreatic pseudocysts are collections of pancreatic fluid (enzymes), surrounded by a fibrous capsule lacking an epithelial lining, which can be located either inside or outside the pancreas.

Several mechanisms may be involved in the formation of a pancreatic pseudocyst, including chronic obstruction of the pancreatic duct with subsequent formation of retention cysts and leakage of pancreatic enzymes [3].

According to Tyberg, spontaneous resolution is common particularly in its early phase, progression of the pseudocyst may culminate in grave consequences such as bleeding, infections, or rupture; our patient presented with an infected, complicated pseudocyst [4].

Pancreatic perirenal pseudocyst is a rare condition that has been only described in literature as case reports before 2020.

In a series of eight patients treated for renal pseudocysts, Rana *et al*. [5] reported normal renal function tests with normal urinary amylase values. In our case, renal function was normal, while urinary amylase was not measured.

Pancreatic perirenal pseudocyst (PPRP) is generally asymptomatic and mainly revealed as incidentally detected renal masses. In some cases, patients can present with acute complications such as perirenal abscess, obstructive hydronephrosis, pseudoaneurysm, or renin mediated hypertension.

Our patient, however, presented with febrile left flank pain.

According to Rana* et al*., endoscopic ultrasound (EUS) is able to demonstrate communication of the main pancreatic duct with the RP in 42% of the patients with chronic pancreatitis [5]. Although the renal pseudocysts (RPs) were well documented on abdominal CT and appeared as perirenal cystic lesions in our case.

In patients who present a diagnostic dilemma, cyst fluid can be aspirated and analyzed for fluid amylase values [6, 7]. In the case of our patient, CT showed collection of the upper pole of the left kidney.

There are no clear guidelines for the management of perirenal pancreatic pseudocysts due to the scarcity of cases. With prompt and appropriate treatment, most people make a full recovery. Surgical or percutaneous drainage are both possible, which can be followed by endoscopic transpapillary drainage with excellent long-term results [8].

We performed percutaneous aspiration to confirm and successfully treat the pancreatic perirenal pseudocyst.

## Conclusion

This is a case report of a 46-year-old North African patient with a history of acute pancreatitis who was initially treated in our urology department for a left kidney abscess, which turned out to be an infected left pararenal pancreatic pseudocyst that progressed well after percutaneous drainage and effective antibiotic treatment.

The aim of our publication was to remind that when a patient with history of pancreatitis presents with fever and lumber pain, an infected perirenal pancreatic pseudocyst should be considered as a possible cause.

Careful clinical examination and imaging modalities, such as CT, may assist in making this difficult diagnosis. Percutaneous drainage and effective antimicrobial treatment is the ideal therapy for this condition.

## Data Availability

The datasets are available from the corresponding author on reasonable request.

## References

[1] Bhasin DK, Rana SS, Nanda M, Chandail VS, Masoodi I, Kang M, Kalra N, Sinha SK, Nagi B, Singh K (2010). Endoscopic management of pancreatic pseudocysts at atypical locations. Surg Endosc.

[2] Ameuraoui T, Alami B, Boubbou M, Maaroufi M, Kamaoui I, Tizniti S (2014). A pseudo-pancreatic cyst mimicking a pancreatic tumor complicated by digestive compression and bleeding: acute pancreatitis, the saga continues. Pan Afr Med J.

[3] Chandraprakash, Mahesh G, Rupinder M, Umesha B, Thimmaiah T, Sherif E, Abhilash P, Hemant G, Shreyas S (2020). Pancreatic fluid collections: clinical manifestations, diagnostic evaluation and management. Dis Mon.

[4] Tyberg A, Karia K, Gabr M, Desai A, Doshi R, Gaidhane M (2016). Management of pancreatic fluid collections: a comprehensive review of the literature. World J Gastroenterol.

[5] Rana SS, Dawra S, Sharma R, Kang M, Gupta R (2020). Clinical manifestations, imaging features, and endoscopic management of renal pseudocysts: a case series. Ann Gastroenterol.

[6] Thandassery RB, Mothilal SK, Singh SK, Khaliq A, Kumar L, Kochhar R, Singh K, Kochhar R (2011). Chronic calcific pancreatitis presenting as an isolated left perinephric abscess: a case report and review of the literature. JOP J Pancreas.

[7] Torres WE, Evert MB, Baumgartner BR, Bernardino ME (1986). Percutaneous aspiration and drainage of pancreatic pseudocysts. AJR Am J Roentgenol.

[8] Heider R, Meyer AA, Galanko JA, Behrns KE (1999). Percutaneous drainage of pancreatic pseudocysts is associated with a higher failure rate than surgical treatment in unselected patients. Ann Surg.

